# Supplementary material for: Transcriptomic evidence for involvement of reactive oxygen species in Rhizoctonia solani AG1 IA sclerotia maturation
Source: PeerJ. 2018 Jun 18;6:e5103. doi: 10.7717/peerj.5103 (PMC6011819; doi:10.7717/peerj.5103)
Supplement: Supplemental Information 1 [file peerj-06-5103-s001.docx]

Table S1. Primers used for RT-qPCR.

| Primer | Seq (5'to3') | PCR efficiency (%) | Regression Coefficient (R^2^) |
| --- | --- | --- | --- |
| 18S-F | ACAAGGATAGTCGCGAGTGG | 104.8 | 0.9985 |
| 18S-R | TGGGCACTGACTCTTTACCA |  |  |
| Actin-F | TATCGTTCTCGATTCTGGTGA | 110.2 | 0.9996 |
| Actin-R | TCAAATCACGACCTGCAAGAT |  |  |
| TUB-F | CTTTCCCTCGTTTGCACTTCT | 104 | 0.9991 |
| TUB-R | CATTTGTTGGGTGAGTTCAGG |  |  |
| GADPH-F | CTATCGCTCAGGCCATTGTT | 98.3 | 0.9975 |
| GADPH-R | ATGCGTCGATCTCCTTGTG |  |  |
| 6PGD-F | TGGGTGAAAGGGACTTCTTG | 102.1 | 0.9971 |
| 6PGD-R | GCTATCGTCGTCGGTATGGT |  |  |
| CLOS7-F | CTTCGCGTGTTCGAGTTGTA | 95.1 | 0.9903 |
| CLOS7-R | CAGCAATCCAGTCTTCACCA |  |  |
| 1.3-β-glucan-F | GTACTTCCTCAAGGCCATCG | 99.8 | 0.9962 |
| 1.3-β-glucan-R | ATTGACCTGACCACGCTTTC |  |  |
| β-galactosidase-F | CCCAAATTCTCCGAGATCAA | 95.5 | 0.9985 |
| β-galactosidase-R | TGGGTTGTGAAGATCTGCTG |  |  |
| NADH dehydrogenase -F | TCTTGATCGGGAATCACACA | 104.2 | 0.9957 |
| NADH dehydrogenase -R | TGAGCGAGTGTATCCGAATG |  |  |
| nadph-p450-F | GGATGCTGTCGTTTGTGATG | 99.2 | 0.9942 |
| nadph-p450-R | CTCAAACCTGATCGGCTCTC |  |  |
| cyp51-F | CAGGTTCCTGAGTGGAAGGA | 97.2 | 0.9824 |
| cyp51-R | GTGGCGGAATCTTTGAGAAC |  |  |
| SOD-F | GGCGAATCCAAAGTTGACAT | 109.5 | 0.9951 |
| SOD-R | GCATGGCCAGTAGTGAGTGA |  |  |
| pg91-F | GTCGCCAACTGGACCTGTAT | 94.9 | 0.9992 |
| pg91-R | AAGGAACACCAGGCACTCAC |  |  |
| 02591-F | GGGTATTGGTGCGACTTTGT | 106.1 | 0.9992 |
| 02591-R | GGGACCTTGTGAGAGAGCAG |  |  |
| chitin synthase-F | ACCCGCTTGGTATGTTTGAC | 100.9 | 0.9996 |
| chitin synthase-R | ATGGATTCTGTGGGCTTGTC |  |  |
| met16-F | AATTACCACGTCGCCATCTC | 101 | 0.9956 |
| met16-R | TACATGCATGGTGCTTTGGT |  |  |

Table S2. Average stability values (SV) of the four candidate reference genes are shown for three stages of *R. solani* AG1 IA.

| Rank | NormFinder | | geNorm | | BestKeeper | | deltaCt | |
| --- | --- | --- | --- | --- | --- | --- | --- | --- |
|  | Gene | SV | Gene | SV | Gene | SV | Gene | SV |
| 1 | 18S | 0.278 | 18S | 0.555 | 18S | 0.463 | 18S | 0.841 |
| 2 | Actin | 0.516 | Actin | 0.555 | GADPH | 0.517 | Actin | 0.97 |
| 3 | GADPH | 0.962 | GADPH | 0.803 | Actin | 0.739 | GADPH | 1.17 |
| 4 | TUB | 1.269 | TUB | 1.089 | TUB | 0.774 | TUB | 1.38 |

Table S3. Top 20 genes in subcluster 1.

| Gene ID | RWF9SIvsRWF9M  log2 Fold Change | RWF9SvsRWF9SI  log2 Fold Change | Putative function |
| --- | --- | --- | --- |
| AG1IA_07154 | -8.444 | -8.5752 | Chitin deacetylase |
| AG1IA_07285 | -8.4268 | -7.7306 | Subtilisin-like protease |
| Novel00787 | -5.8562 | -7.1819 | Probable pectate lyase E |
| AG1IA_03265 | -5.8281 | -6.8682 | Beta-glucan synthesis-associated protein KRE6 |
| AG1IA_08241 | -6.1224 | -6.295 | Uncharacterized glycosidase |
| AG1IA_04672 | -6.2147 | -6.2716 | Laccase-2 |
| Novel01102 | -7.6402 | -6.2233 | Sterol reductase |
| Novel00616 | -5.3048 | -6.0015 | Extracellular metalloprotease SMAC_06893 |
| AG1IA_05701 | -6.3566 | -5.7589 | C-8 sterol isomerase |
| Novel01896 | -5.2045 | -5.3916 | Manganese peroxidase 2 |
| Novel01553 | -5.6473 | -5.2684 | Meiotic recombination protein dmc1 |
| Novel00400 | -5.0823 | -5.0498 | Mannosyl-oligosaccharide alpha-1,2-mannosidase 1B |
| Novel00647 | -2.7811 | -4.9749 | Obtusifoliol 14-alpha demethylase (Fragment) |
| Novel01544 | -4.1004 | -4.9598 | Leucine aminopeptidase 1 |
| Novel01152 | -4.2515 | -4.9144 | Delta(14)-sterol reductase |
| Novel01651 | -3.2278 | -4.6808 | Peptidyl-Lys metalloendopeptidase |
| Novel00184 | -3.1595 | -4.6927 | Benzoate 4-monooxygenase |
| AG1IA_09055 | -4.4068 | -4.6944 | Pathogenesis-related protein 5 |
| Novel00898 | -4.0446 | -4.7164 | Uncharacterized protein YpgQ OS=Bacillus subtilis |
| AG1IA_06618 | -2.6284 | -4.3966 | rhamnogalacturonate lyase A |
| AG1IA_09287 | -4.015 | -3.5331 | Glucan 1,3-beta-glucosidase |

Table S4. Top 20 genes in subcluster 2.

| Gene ID | RWF9SIvsRWF9M  log2 Fold Change | RWF9SvsRWF9SI  log2 Fold Change | Putative function |
| --- | --- | --- | --- |
| AG1IA_07062 | 3.1283 | -4.7638 | Polysaccharide monooxygenase |
| AG1IA_05259 | 2.7288 | -3.846 | mitotic-specific cyclin |
| Novel00097 | 3.3395 | -3.5994 | Thymocyte nuclear protein 1 |
| AG1IA_08887 | 2.668 | -2.9637 | Protein priA |
| AG1IA_02048 | 2.326 | -2.6384 | Serine/threonine-protein kinase |
| AG1IA_07526 | 1.7658 | -2.7685 | Formate dehydrogenase |
| Novel00314 | 1.9661 | -2.8307 | ATP-dependent DNA helicase II subunit 2 |
| AG1IA_01597 | 2.2917 | -2.561 | mitotic-specific cyclin |
| AG1IA_04347 | 1.9833 | -2.4681 | Histone H2A |
| AG1IA_05662 | 1.5395 | -2.3286 | Histone H4 |
| AG1IA_00180 | 2.0403 | -2.2472 | Peptide-N4-(N-acetyl-beta-glucosaminyl)asparagine amidase A |
| AG1IA_04350 | 2.2922 | -2.0802 | Histone H32 |
| AG1IA_00561 | 2.2449 | -2.0722 | Carboxylesterase 1D |
| AG1IA_07010 | 1.8286 | -2.0246 | Serine/threonine-protein kinase ark1 |
| AG1IA_01030 | 1.5695 | -2.0365 | HMG box-containing protein |
| AG1IA_08165 | 1.4399 | -1.9409 | Farnesylcysteine lyase |
| AG1IA_03646 | 2.3055 | -1.927 | Cellulose-growth-specific protein |
| AG1IA_02513 | 1.031 | -1.8775 | Chitin deacetylase |
| AG1IA_02659 | 1.576 | -1.5628 | 1,3-beta-glucanosyltransferase gel4 |
| AG1IA_01287 | 2.6098 | -1.8158 | Plasma membrane fusion protein PRM1 |
| AG1IA_07633 | 1.0644 | -1.6178 | Mannosyl-oligosaccharide 1,2-alpha-mannosidase |

Table S5. Top 20 genes in subcluster 3.

| Gene ID | RWF9SIvsRWF9M  log2 Fold Change | RWF9SvsRWF9SI  log2 Fold Change | Putative function |
| --- | --- | --- | --- |
| AG1IA_08432 | -6.1236 | -3.0569 | Cytochrome P450 |
| AG1IA_07609 | -6.4456 | -3.2968 | Phosphoadenosine phosphosulfate reductase |
| AG1IA_06812 | -6.5253 | -3.3036 | Protein NMT1 homolog |
| AG1IA_02916 | -5.5676 | -3.3567 | Meiotically up-regulated gene 157 protein |
| AG1IA_03335 | -5.1526 | -2.8606 | Iron transporter FTH1 |
| Novel01882 | -5.7267 | -2.8775 | O-methylsterigmatocystin oxidoreductase |
| Novel00515 | -6.9353 | -2.4306 | Carboxyvinyl-carboxyphosphonate phosphorylmutase |
| AG1IA_06996 | -6.8103 | -2.434 | Trans-aconitate 3-methyltransferase |
| AG1IA_00722 | -4.73 | -2.1637 | Alkaline phosphatase |
| AG1IA_07255 | -4.9205 | -2.3701 | Killer toxin subunits alpha/beta |
| AG1IA_08022 | -5.2223 | -1.873 | 1-aminocyclopropane-1-carboxylate synthase-like protein 1 |
| Novel01209 | -7.8416 | -1.9004 | Putative hydrolase |
| Novel01731 | -4.8428 | -1.6653 | Chitinase 1 |
| AG1IA_01905 | -4.5798 | -1.694 | Phenol 2-monooxygenase |
| AG1IA_04807 | -4.3271 | -1.5588 | Uncharacterized aminotransferase |
| AG1IA_08900 | -4.489 | -1.3637 | 6-hydroxynicotinate 3-monooxygenase |
| AG1IA_06854 | -3.6278 | -1.2349 | Uncharacterized oxidoreductase |
| AG1IA_06304 | -3.8096 | -1.1749 | Calpain |
| AG1IA_08342 | -4.382 | -1.1852 | Protein ura1 |
| AG1IA_04666 | -3.3486 | -1.1822 | 2-dehydro-3-deoxy-D-gluconate 5-dehydrogenase |

Table S6. Top 20 genes in subcluster 4.

| Gene ID | RWF9SIvsRWF9M  log2 Fold Change | RWF9SvsRWF9SI  log2 Fold Change | Putative function |
| --- | --- | --- | --- |
| AG1IA_08569 | 5.3085 | 4.6058 | Obtusifoliol 14-alpha demethylase |
| Novel00113 | 1.7107 | 4.4422 | Exoglucanase 1 |
| Novel00851 | 4.6911 | 3.4653 | Alcohol dehydrogenase 3 |
| AG1IA_05123 | 3.7307 | 3.2908 | L-2,4-diaminobutyrate decarboxylase |
| AG1IA_03752 | 1.4766 | 2.8924 | Meiotically up-regulated gene 190 protein |
| AG1IA_02247 | 1.926 | 2.832 | O-methylsterigmatocystin oxidoreductase |
| Novel01824 | 3.0922 | 2.5805 | mannose 3,5-epimerase 1 |
| AG1IA_05540 | 3.048 | 2.5999 | Rhamnolipids biosynthesis 3-oxoacyl-[acyl-carrier-protein] reductase |
| AG1IA_03751 | 1.7467 | 2.6008 | Endo-1,4-beta-xylanase C |
| AG1IA_10228 | 2.3249 | 2.1912 | NADPH-P450 reductase |
| AG1IA_07900 | 1.3463 | 2.0661 | Cys-Gly metallodipeptidase dug1 |
| AG1IA_08773 | 1.0285 | 5.5589 | Catalase |
| AG1IA_03629 | 2.1281 | 2.0066 | NADPH-P450 reductase |
| AG1IA_03770 | 1.7434 | 1.8767 | aldehyde dehydrogenase |
| Novel01569 | 3.8093 | 1.899 | Ras-related protein RABB1b |
| AG1IA_03184 | 1.4703 | 1.7462 | Rhamnogalacturonan acetylesterase |
| Novel00615 | 5.239 | 1.72 | endopolygalacturonase A |
| AG1IA_01301 | 1.3729 | 1.6256 | O-methyltransferase MdmC |
| AG1IA_07837 | 1.7746 | 1.5447 | Peptidyl-prolyl cis-trans isomerase slr1251 |
| Novel01852 | 1.666 | 1.5593 | O-methylsterigmatocystin oxidoreductase |

Table S7. Top 20 genes in subcluster 5.

| Gene ID | RWF9SIvsRWF9M  log2 Fold Change | RWF9SvsRWF9SI  log2 Fold Change | Putative function |
| --- | --- | --- | --- |
| Novel00973 | 2.1313 | 6.3238 | Exoglucanase 1 |
| Novel00952 | 1.1138 | 5.6155 | Uncharacterized protein |
| Novel01616 | -1.9559 | 5.6651 | Putative amidase |
| Novel00860 | -1.3564 | 5.7503 | Putative magnesium-dependent phosphatase |
| AG1IA_06235 | -1.14 | 4.9916 | methyltransferase erg6 |
| AG1IA_08531 | -2.8081 | 5.0432 | Aliphatic nitrilase |
| Novel00762 | -1.1794 | 4.1706 | Alkali-sensitive linkage protein 1 |
| AG1IA_01763 | -1.621 | 4.1954 | Purine-cytosine permease fcyB |
| AG1IA_00671 | -1.0984 | 4.1444 | ATP-dependent RNA helicase FAL1 |
| AG1IA_10013 | -1.1105 | 4.0183 | Rhamnolipids biosynthesis 3-oxoacyl-[acyl-carrier-protein] reductase |
| AG1IA_01162 | 1.1659 | 4.0313 | Conidiation-specific protein 6 |
| Novel00395 | -2.0409 | 3.8672 | Conidiation-specific protein 6 |
| Novel01085 | -1.4712 | 3.6999 | Endo-beta-1,4-glucanase D |
| AG1IA_05655 | -1.5318 | 3.7318 | Rhamnolipids biosynthesis 3-oxoacyl-[acyl-carrier-protein] reductase |
| AG1IA_07183 | -2.2884 | 3.7539 | Cyanate hydratase |
| AG1IA_05549 | -1.4591 | 3.651 | NADPH-dependent curcumin reductase |
| AG1IA_09843 | -2.424 | 3.6558 | Cytochrome P450 |
| AG1IA_05550 | -1.6809 | 3.681 | Putative NADP-dependent oxidoreductase YfmJ |
| AG1IA_06952 | -1.4589 | 3.6099 | phosphoketolase |
| AG1IA_10295 | -2.3952 | 3.5902 | Steroid 17-alpha-hydroxylase/17,20 lyase |

Table S8. Top 20 genes in subcluster 6.

| Gene ID | RWF9SIvsRWF9M  log2 Fold Change | RWF9SvsRWF9SI  log2 Fold Change | Putative function |
| --- | --- | --- | --- |
| AG1IA_02594 | -6.0966 | 1.3475 | O-methylsterigmatocystin oxidoreductase |
| AG1IA_00637 | -5.5386 | 1.6912 | 3-isopropylmalate dehydrogenase |
| Novel01950 | -5.351 | 1.9253 | Protein TOXD |
| Novel01562 | -5.2866 | -1.0987 | O-methylsterigmatocystin oxidoreductase |
| AG1IA_04232 | -5.2741 | 2.1713 | Homoisocitrate dehydrogenase |
| Novel00826 | -5.0508 | 1.8995 | Cystathionine gamma-lyase |
| Novel01065 | -4.8713 | 1.0122 | Pyridoxal reductase |
| AG1IA_06380 | -4.8087 | 3.2599 | 2-iminobutanoate |
| AG1IA_00128 | -4.7899 | 1.1767 | Glucose-6-phosphate 1-dehydrogenase |
| AG1IA_00217 | -4.7334 | 1.8376 | Heat shock protein 90 |
| AG1IA_00127 | -4.6488 | 1.1494 | Glucose-6-phosphate 1-dehydrogenase |
| Novel00373 | -4.6155 | -1.4544 | GDSL esterase/lipase At1g29670 |
| AG1IA_02434 | -4.5662 | 1.0222 | Argininosuccinate synthase |
| AG1IA_05826 | -4.5144 | 2.1484 | Low-specificity L-threonine aldolase |
| Novel00246 | -4.5103 | -2.0932 | N-acetyltransferase san |
| AG1IA_03830 | -4.4888 | 1.1519 | phosphatase |
| AG1IA_04168 | -4.4833 | 1.4209 | Glucokinase |
| Novel01527 | -4.4642 | 1.3046 | Laccase-1 |
| AG1IA_02400 | -4.3187 | 2.045 | Glucose-6-phosphate isomerase |
| AG1IA_06045 | -4.2556 | 2.0815 | S-formylglutathione hydrolase |

Table S9. Top 20 genes in subcluster 7.

| Gene ID | RWF9SIvsRWF9M  log2 Fold Change | RWF9SvsRWF9SI  log2 Fold Change | Putative function |
| --- | --- | --- | --- |
| AG1IA_06140 | 7.3939 | -6.5817 | Tyrosinase |
| Novel01680 | 7.8469 | -9.1949 | NADPH dehydrogenase |
| AG1IA_04673 | 5.62 | -6.6867 | Laccase-3 |
| Novel00623 | 5.2837 | -4.6464 | Cytochrome P450 |
| AG1IA_10009 | 5.0381 | -3.522 | beta-galactosidase C |
| AG1IA_08834 | 5.077 | -8.7489 | Laccase-5 |
| Novel01862 | 4.9837 | -6.8765 | Aquaporin-7 |
| Novel00674 | 4.212 | -3.1414 | PII-type proteinase |
| Novel00943 | 4.2698 | -6.7576 | Tyrosinase |
| AG1IA_07325 | 4.2708 | -2.7485 | Galactinol synthase 7 |
| AG1IA_02246 | 4.3509 | -3.0265 | E3 ubiquitin-protein ligase ORTHRUS 1 |
| AG1IA_07864 | 3.8042 | -3.0087 | Ammonia transport outward protein 2 |
| Novel00205 | 3.8465 | -3.3124 | Oxalate decarboxylase OxdC |
| AG1IA_02591 | 3.6817 | -3.8479 | Oxygen-dependent choline dehydrogenase |
| AG1IA_02281 | 3.3385 | -3.1905 | D-xylulose kinase A |
| AG1IA_08909 | 3.2703 | -2.6105 | Sexual differentiation process protein isp4 |
| AG1IA_04638 | 3.1055 | -3.0195 | Meiotic expression up-regulated protein 26 |
| Novel01177 | 3.0654 | -5.7152 | Putative pectinesterase 11 |
| AG1IA_07728 | 2.9608 | -2.0335 | Cell division control protein 25 |
| AG1IA_02619 | 2.7552 | -2.1176 | Chitin synthase |

Table S10. Top 20 genes in subcluster 8.

| Gene ID | RWF9SIvsRWF9M  log2 Fold Change | RWF9SvsRWF9SI  log2 Fold Change | Putative function |
| --- | --- | --- | --- |
| Novel01355 | 16.186 | -8.1154 | hypothetical protein |
| Novel01360 | 14.777 | -7.2954 | hypothetical protein |
| AG1IA_10480 | 13.923 | -7.3358 | Oxygen-dependent choline dehydrogenase |
| Novel01696 | 12.798 | -4.0708 | hypothetical protein |
| AG1IA_10043 | 11.871 | -8.537 | hypothetical protein |
| Novel00963 | 11.313 | -2.0024 | Uncharacterized membrane protein YFL054C |
| Novel01597 | 10.16 | 1.0311 | hypothetical protein |
| Novel00210 | 10.066 | 1.0018 | hypothetical protein |
| Novel01993 | 10.064 | -6.9581 | Alcohol dehydrogenase |
| AG1IA_02934 | 9.7836 | 1.1092 | hypothetical protein |
| AG1IA_08167 | 9.6049 | -9.9587 | hypothetical protein |
| Novel00845 | 9.5802 | 1.8006 | hypothetical protein |
| Novel01942 | 9.3886 | -6.8921 | hypothetical protein |
| AG1IA_08901 | 9.2624 | 1.5475 | ricin-type beta-trefoil lectin domain-containing protein |
| AG1IA_04772 | 8.7893 | 2.2291 | peptidase inhibitor clitocypin domain-containing protein |
| Novel00350 | 8.7071 | 1.7747 | Ammonia transport outward protein 2 |
| AG1IA_02407 | 8.5438 | 2.605 | hypothetical protein |
| AG1IA_01640 | 7.9806 | 1.0157 | Ammonium transporter |
| AG1IA_08824 | 7.4376 | -1.0052 | hypothetical protein |
| AG1IA_01639 | 7.3831 | 1.1528 | Ammonium transporter 1 |
