# Supplementary material for: Transcriptomic evidence for involvement of reactive oxygen species in Rhizoctonia solani AG1 IA sclerotia maturation
Source: PeerJ. 2018 Jun 18;6:e5103. doi: 10.7717/peerj.5103 (PMC6011819; doi:10.7717/peerj.5103)
Supplement: Supplemental Information 2 [file peerj-06-5103-s002.docx]

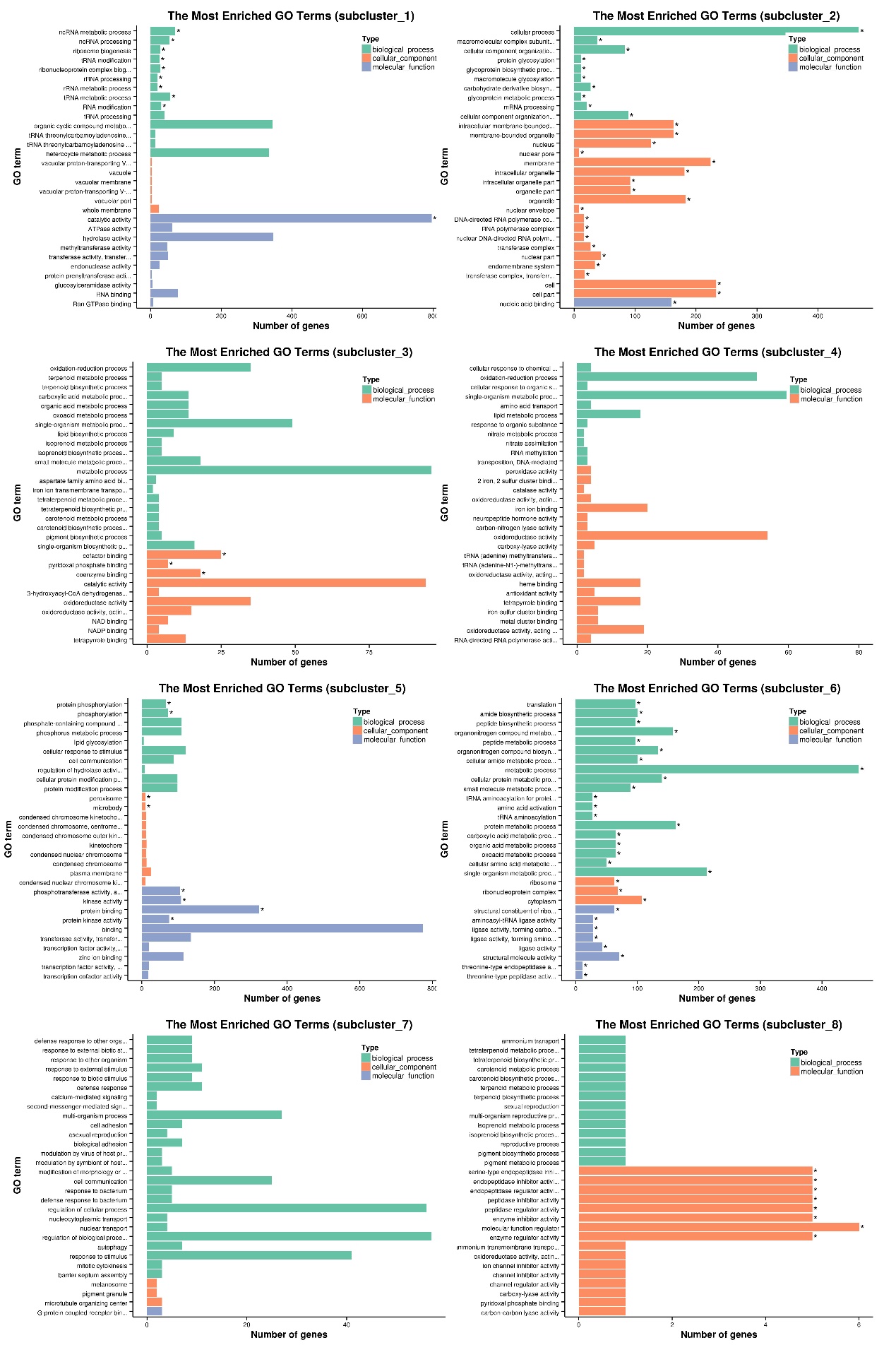


Fig. S1 The difference of GO gene enrichment column (eight cluster), which reflects in the biological process, cellular component and molecular function. The ordinate is the enrichment of GO term, the abscissa is the number of genes in the term. In different colors to distinguish between biological processes, cell component and molecular function.


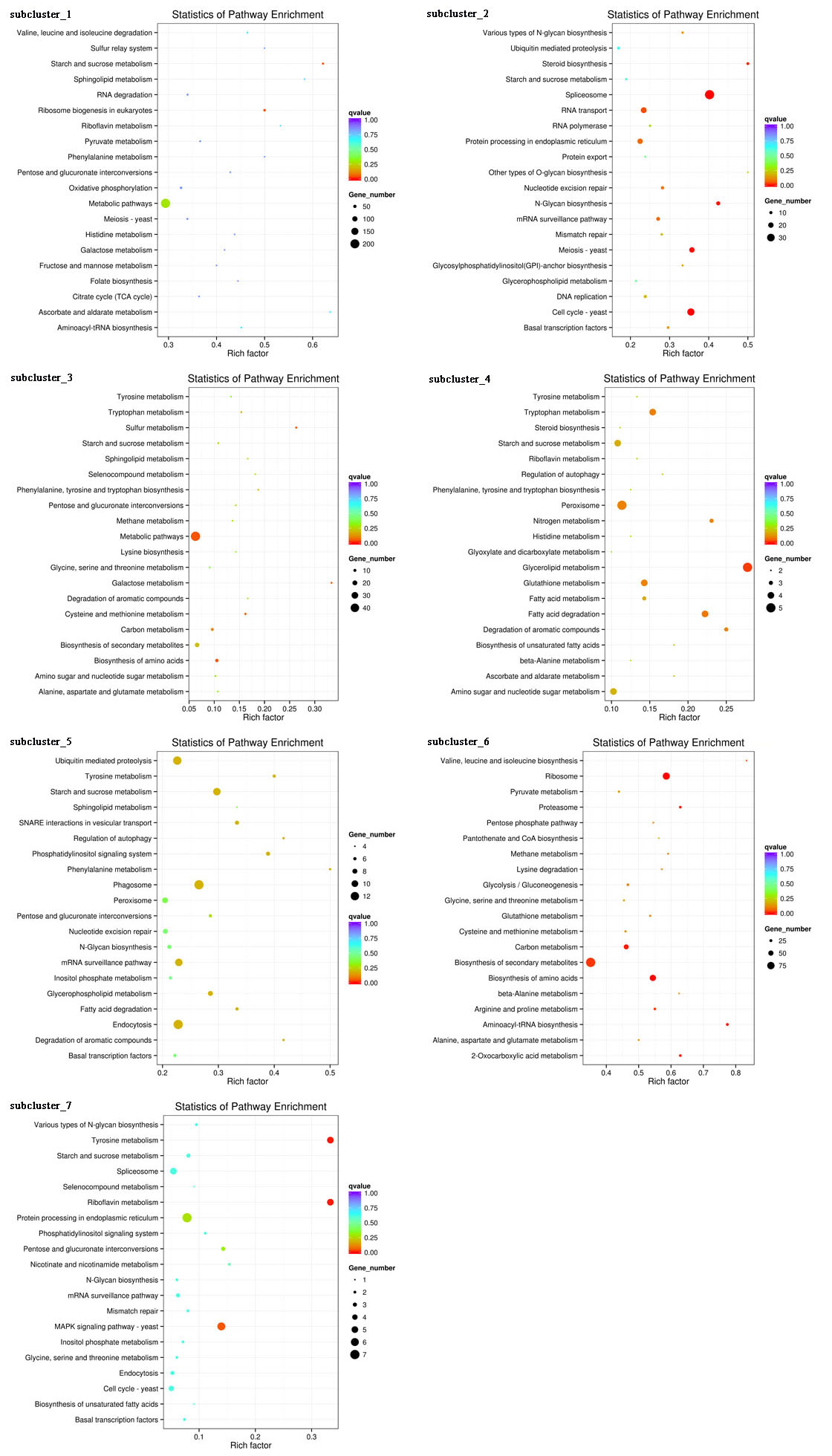


Fig. S2 Statistics of Pathway Enrichment (seven cluster). The ordinate is the pathway, the abscissa is the Rich factor. The size of the point represents the number of differentially expressed genes in this pathway, and the color of the point corresponds to the different Q-value ranges.


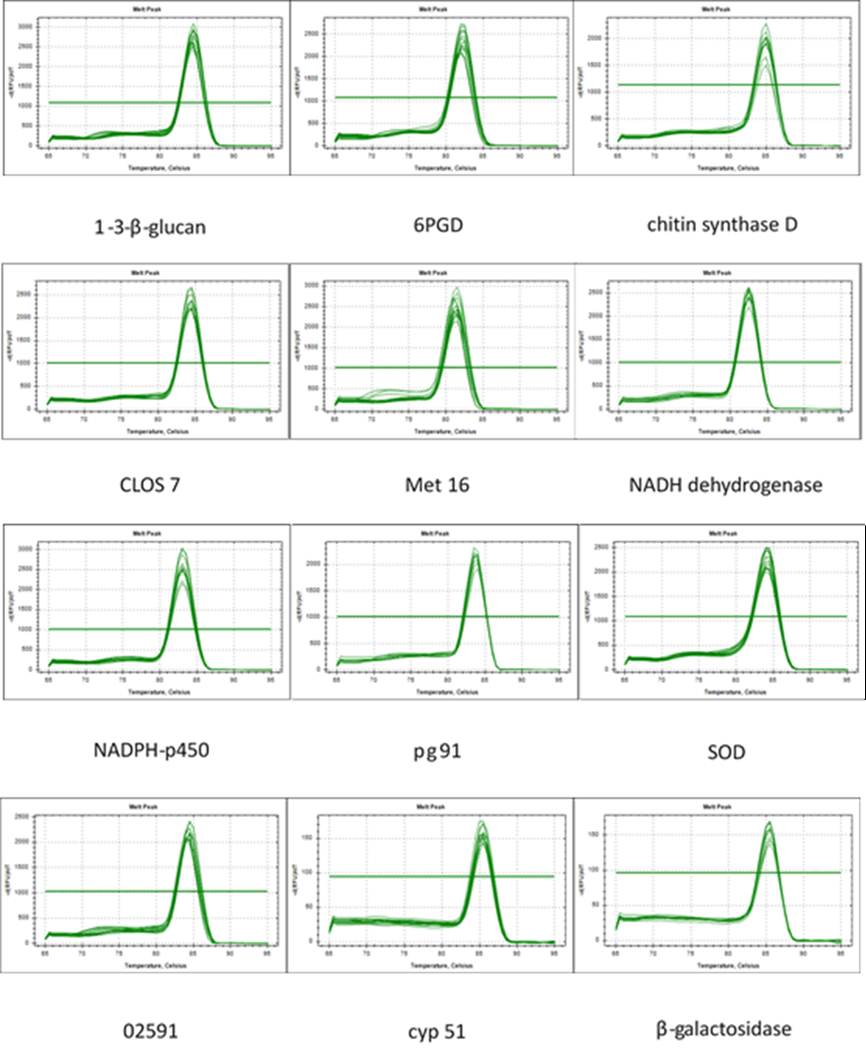


Fig. S4 Treated the samples with the CAT inhibitor aminotriazole.

Fig. S3 Treated the samples with H_2_O_2_.


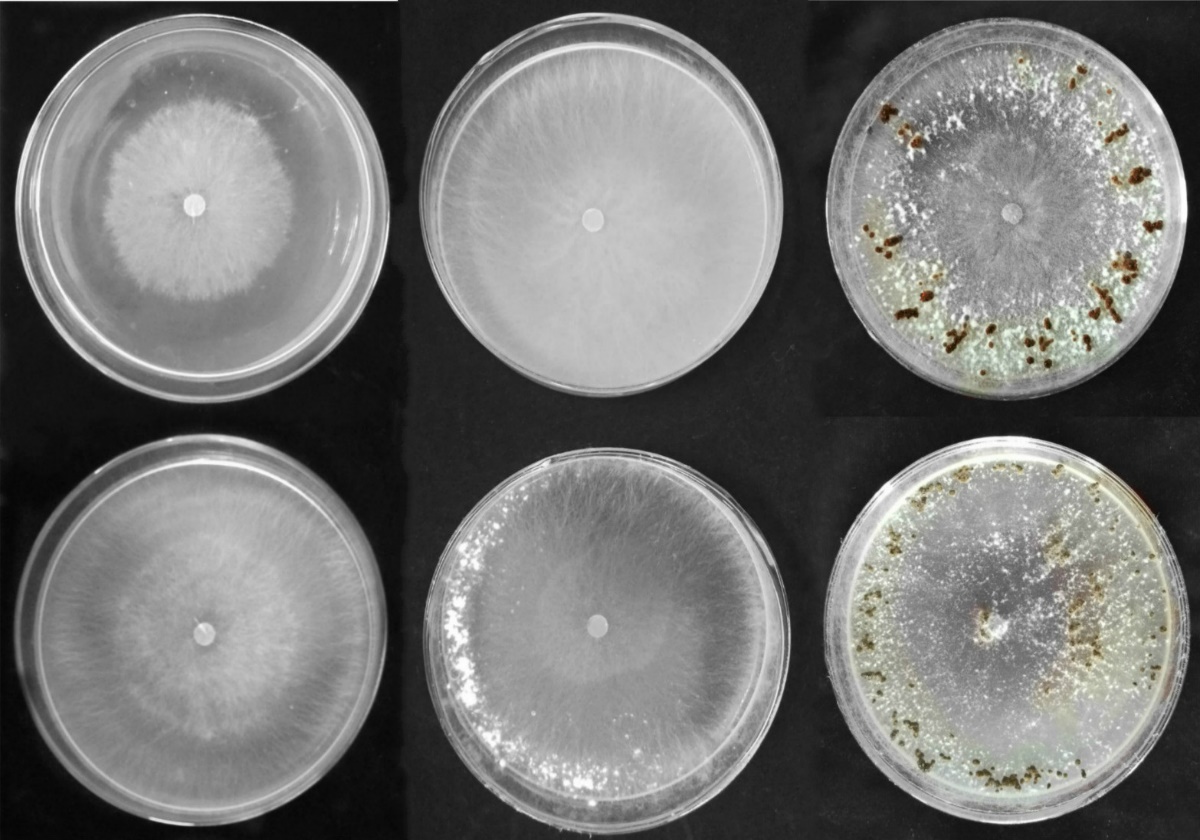


2 days

3 days

7 days

CK

H_2_O_2_


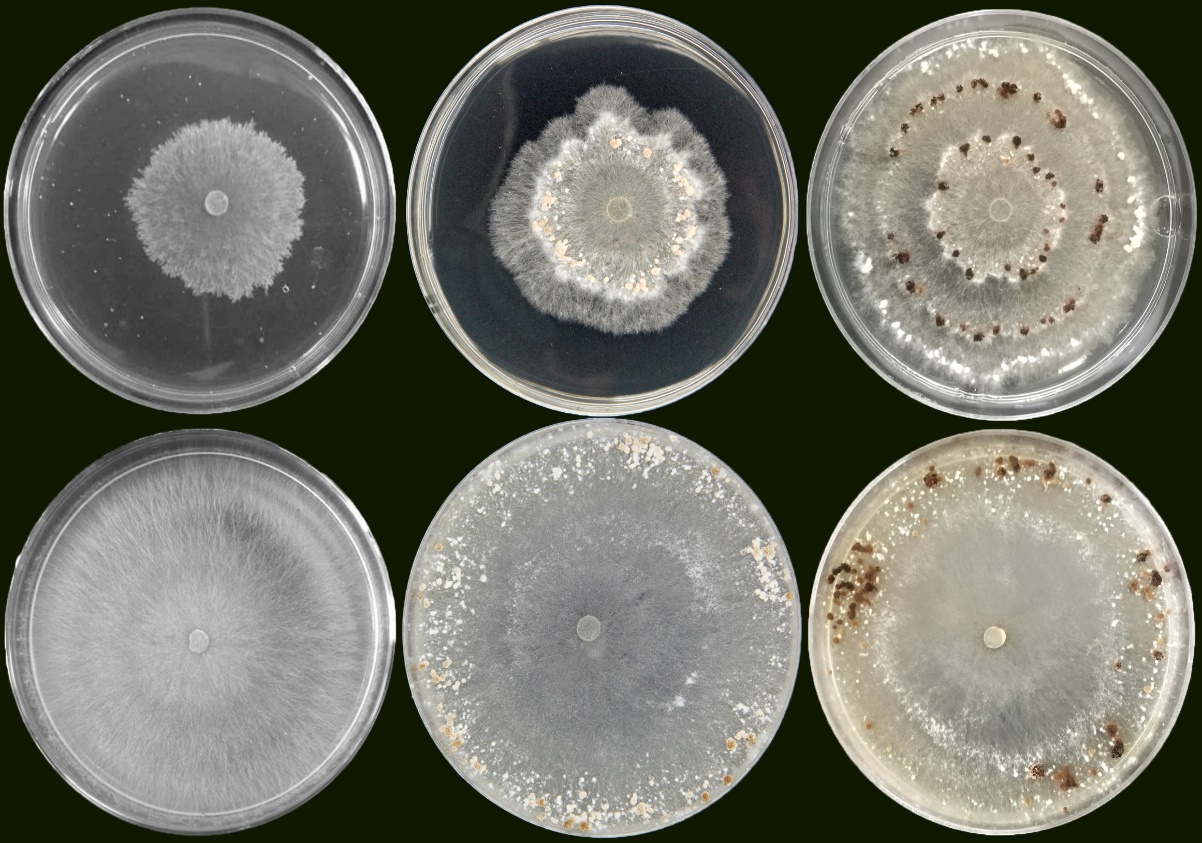


3 days

4 days

7 days

aminotriazole

CK

Fig. S5 Melting curve for twelve candidate genes ((*1.3-β-glucan*) glucan 1,3-beta-glucosidase, (*6PGD*) 6-phosphogluconate dehydrogenase, chitin synthase D, (*CLOS7*) galactinol synthase 7, (*Met16*) phosphoadenosine phosphosulfate reductase, NADH dehydrogenase, (*NADPH-P450*) NADPH-P450 reductase, (*pg91*) NADPH oxidases, (*SOD*) superoxide dismutase, (02591) oxygen-dependent choline dehydrogenase, (*CYP51*) obtusifoliol 14-alpha demethylase, beta-galactosidase) with single peak obtained from three technical replicates of different cDNA pools along with no template control.

**300bp**

**200bp**

**100bp**


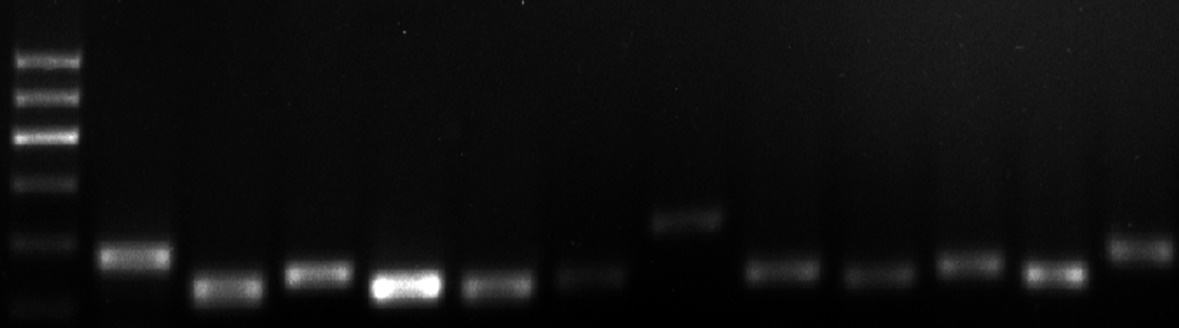


**M**

**NADH**

**pg91**

**cyp51**

**Met16**

**CLOS7**

**NADPH -P450**

**02591**

**β-galactosidase**

**SOD**

**6PGD**

**chitin**

**1,3-β-glucan**

Fig. S6 Agarose gel electrophoresis showing specific RT-PCR products of the expected size for each reference gene obtained by real-time PCR using cDNA as template. (M) DNA marker; (*NADH*) NADH dehydrogenase; (*pg91*) NADPH oxidases; (*CYP51*) obtusifoliol 14-alpha demethylase; (*Met16*) phosphoadenosine phosphosulfate reductase; (*CLOS7*) galactinol synthase 7; (*NADPH-P450*) NADPH-P450 reductase; (02591) oxygen-dependent choline dehydrogenase; beta-galactosidase; (*SOD*) superoxide dismutase; (*1.3-β-glucan*) glucan 1,3-beta-glucosidase; (*6PGD*) 6-phosphogluconate dehydrogenase; (*chitin*) chitin synthase D)


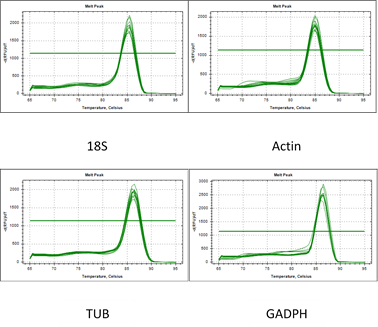


Fig. S7 Melting curve for four candidate reference genes ((*18S*) 18S ribosomal RNA, Actin, (*TUB*) β-tubulin; (*GADPH*) glyceraldehyde-3-phosphate dehydrogenase) with single peak obtained from three technical replicates of different cDNA pools along with no template control.


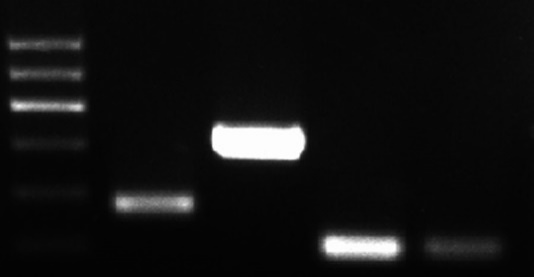


**100bp**

**200bp**

**300bp**

**Actin**

**TUB**

**GADPH**

**18S**

**M**

Fig. S8 Agarose gel electrophoresis showing specific RT-PCR products of the expected size for each reference gene obtained by real-time PCR using cDNA as template. (M) DNA marker; (*18S*) 18S ribosomal RNA; (*GADPH*) glyceraldehyde-3-phosphate dehydrogenase; (*TUB*) β-tubulin; (*Actin*) Actin.


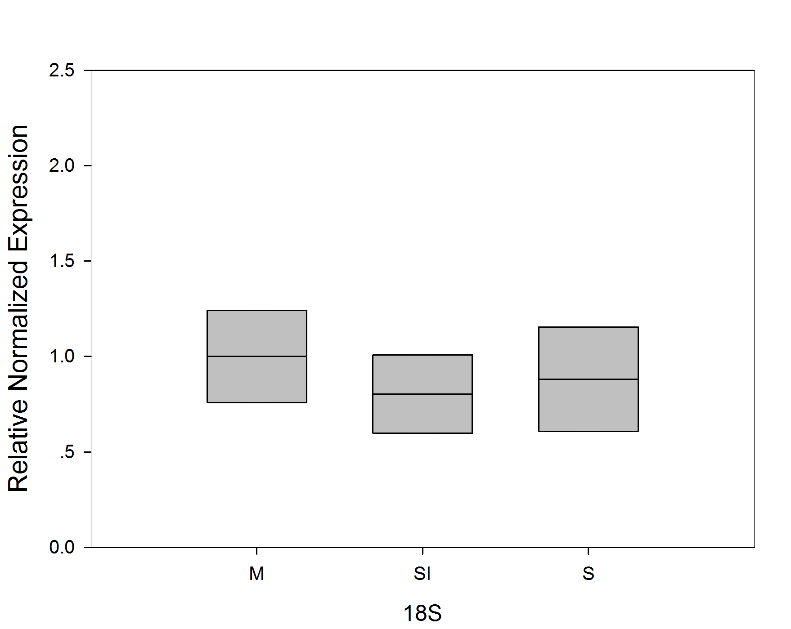


Fig. S9 Actin was reference, expressions of 18S in three stages of *R. solani* AG1 IA.
